# ARE RURAL OLDER VETERANS SATISFIED WITH VIRTUAL COMPREHENSIVE GERIATRIC ASSESSMENTS?

**DOI:** 10.1093/geroni/igad104.3181

**Published:** 2023-12-21

**Authors:** Linda Sawyer, Hallie Keller, Teresa Howell, Maria Cervantes, Janette Dunlap, Christine Cigolle, Dennis Sullivan

**Affiliations:** Central Arkansas Veterans Healthcare System, North Little Rock, Arkansas, United States; Central Arkansas Veterans Healthcare System, North Little Rock, Arkansas, United States; North Florida/South Georgia Veterans Health System, Gainesville, Florida, United States; Veterans Health Administration, Saint Johns, Florida, United States; North Florida/South Georgia Veterans Health System, Gainesville, Florida, United States; Ann Arbor VAMC, Ann Arbor, Michigan, United States; Central Arkansas Veterans Healthcare System, North Little Rock, Arkansas, United States

## Abstract

Rural older Veterans (ROV) have limited access to comprehensive geriatric assessments (CGA). VA Video Connect (VVC) can potentially overcome this access barrier. A standardized process was developed and piloted, to conduct CGAs by VVC. It was found to be feasible and facilitated access to CGAs. The purpose of this study was to assess ROVs’ satisfaction with CGA via VVC. The team developed a 10-item questionnaire with good face and content validity to assess satisfaction with the CGA and process. All ROVs who completed a CGA via VVC were telephoned. They were asked to rate each item on a Likert scale. Caregivers were allowed to assist but the final answer came from Veteran themselves. The results are summarized as percentage of respondents who “agree” or “strongly agree”. ROVs also provided comments. Of the 79 ROVs who completed the CGA via VVC, 63 OVs completed the questionnaire. Most were white (82.8%) males (95.4%), with mean(±SD) age 75.7(±6.5). Most felt it was easy to communicate with the providers (95.2%) and understood the information they were given (98.4%). Most felt comfortable using the technology (75.8%) and that the visit was private enough (96.8%). Although many commented they would prefer in-person visits, most agreed that they would do virtual visits again if offered (71.4%). Overall, they “agreed that they would not change anything about the CGA process (95.2%). The results suggest that ROVs were satisfied with CGA via VVC. More research is needed to determine the outcomes, benefits, and limitations of CGAs performed by VVC.

